# 9,9-Dioctyl-2,7-bis­(4,4,5,5-tetra­methyl-1,3,2-dioxaborolan-2-yl)-9*H*-fluorene

**DOI:** 10.1107/S1600536808022496

**Published:** 2008-07-26

**Authors:** Eric Gagnon, Dominic Laliberté

**Affiliations:** a Département de Chimie, Université of Montréal, CP 6128, succ. Centre-ville, Montréal, Québec, Canada H3C 3J7; b SolarisChem Inc., 598 Chaline Street, St-Lazare, Québec, Canada J7T 3E8

## Abstract

In the title mol­ecule, C_41_H_64_B_2_O_4_, the fluorene unit is essentially planar and the two octyl chains attached to the central C atom inhibit the mol­ecule from engaging in inter­molecular aromatic inter­actions. One of the octyl chains adopts a fully extended conformation, whereas the second incorporates a single *gauche* conformation. Of the two pinacolatoboronate groups attached at the 2,7-positions, one is partly disordered; one ring C atom and all four methyl groups are disordered equally over two positions.

## Related literature

For related literature, see: Cho *et al.* (2007 ▶); Scherf & List (2002 ▶).
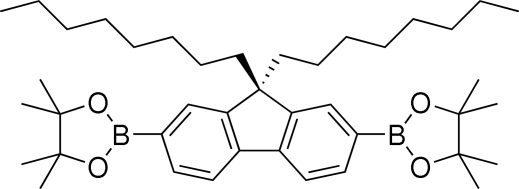


## Experimental

### 

#### Crystal data

C_41_H_64_B_2_O_4_
*M* *_r_* = 642.54
Triclinic, 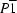

*a* = 12.6694 (12) Å
*b* = 13.3457 (11) Å
*c* = 14.0819 (11) Å
α = 68.944 (3)°
β = 89.834 (4)°
γ = 64.306 (4)°
*V* = 1968.9 (3) Å^3^
*Z* = 2
Cu *K*α radiation
μ = 0.51 mm^−1^
*T* = 150 K
0.10 × 0.10 × 0.05 mm

#### Data collection

Bruker Microstar diffractometer
Absorption correction: multi-scan (*SADABS*; Sheldrick, 2007 ▶) *T*_min_ = 0.840, *T*_max_ = 0.975
30971 measured reflections
6210 independent reflections
5656 reflections with *I* > 2σ(*I*)
*R*_int_ = 0.054

#### Refinement

*R*[*F* ^2^ > 2σ(*F* ^2^)] = 0.039
*wR*(*F* ^2^) = 0.104
*S* = 1.04
6210 reflections
476 parameters
66 restraints
H-atom parameters constrained
Δρ_max_ = 0.24 e Å^−3^
Δρ_min_ = −0.18 e Å^−3^

### 

Data collection: *APEX2* (Bruker, 2006 ▶); cell refinement: *SAINT* (Bruker, 2006 ▶); data reduction: *SAINT*; program(s) used to solve structure: *SHELXS97* (Sheldrick, 2008 ▶); program(s) used to refine structure: *SHELXL97* (Sheldrick, 2008 ▶); molecular graphics: *SHELXTL* (Sheldrick, 2008 ▶) and *Materials Studio* (Accelrys, 2005 ▶); software used to prepare material for publication: *UdMX* (Maris, 2004 ▶).

## Supplementary Material

Crystal structure: contains datablocks global, I. DOI: 10.1107/S1600536808022496/lh2661sup1.cif

Structure factors: contains datablocks I. DOI: 10.1107/S1600536808022496/lh2661Isup2.hkl

Additional supplementary materials:  crystallographic information; 3D view; checkCIF report

## Figures and Tables

**Fig. 1.**
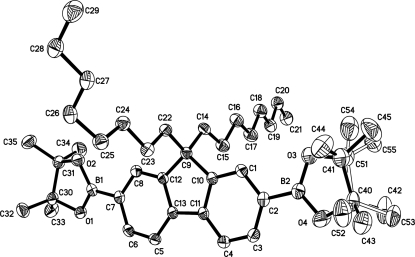
A view of the molecular structure showing the disorder. Displacement ellipsoids are drawn at the 50% probability level. Hydrogen atoms are omitted for clarity.

**Fig. 2.**
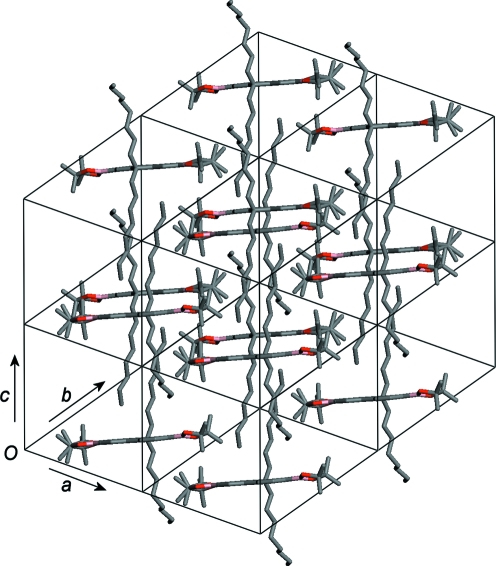
A view of a 2x2x2 array of unit cells showing the coplanar fluorene units and the perpendicular octyl groups. Hydrogen atoms are omitted for clarity.

## supplementary crystallographic information

### Comment

Fluorene derivatives have found many applications in chemistry, especially in the optoelectronic area. Polymers based on the 9,9-dialkylfluorene motif possess good thermal stability along with interesting emissive properties. The quality and efficiency of OLEDs and sensors using thin films of these polymers have been shown to depend critically on the stacking of the molecules. The film-forming properties can be tailored by a judicious choice of alkyl chains, be it *n*-alkyl of different lengths or other branched alkyl chains. The selected alkyl groups have a profound effect on the solubility and the packing of oligo- and polyfluorenes (Scherf & List, 2002). During the process of developing new polymers, we were able to crystallize the title compound from THF/methanol.

The two alkyl chains behave quite differently in the crystal. One of them adopts a fully extended conformation with torsional angles ranging from 173.52 (12)° to 179.74 (12)°. The second octyl group incorporates a single *gauche* conformation (C24—C25—C26—C27, torsional angle: 70.95 (15)°), and the other torsional angles range from 171.05 (11)° to 179.67 (12)°.

In the crystal, the fluorene units are coplanar with each other and the octyl chains are extended perpendicular to the aromatic plane. The fluorene units are thereby isolated from one another by the octyl groups, as well as the pinacol groups, and no π-π interactions are present.

### Experimental

The title compound was prepared according to Cho *et al.* (2007) from the corresponding 2,7-dibromo-9,9-dioctylfluorene. Purified material was obtained by recrystallization from THF/methanol. Spectroscopic data are consistent with the reported values.

### Refinement

Non-H atoms were refined anisotropicaly. H atoms were placed in idealized positions and allowed to ride on their parent atoms with C—H distances of 0.98 Å (methylene), 0.99 Å (methyl), and 0.95 Å (aromatic C—H) and with *U*_iso_ of 1.2 times *U*_eq_(C) for aromatic and methylene H atoms and 1.5 times *U*_eq_(C) for terminal methyl groups. One of the pinacolatoboronate moieties is disordered over two positions in a 1:1 ratio as determined crystallographically. All the C—CH_3_ bonds in the disordered and the non-disordered pinacolatoboronates were restrained to be of similar length (SADI restraints with default standard deviations).

### Crystal data

**Table:**

C_41_H_64_B_2_O_4_	*Z* = 2
*M**_r_* = 642.54	*F*_000_ = 704
Triclinic, *P*1	*D*_x_ = 1.084 Mg m^−^^3^
Hall symbol: -P 1	Cu *K*α radiation λ = 1.54178 Å
*a* = 12.6694 (12) Å	Cell parameters from 17842 reflections
*b* = 13.3457 (11) Å	θ = 3.4–68.2º
*c* = 14.0819 (11) Å	µ = 0.51 mm^−^^1^
α = 68.944 (3)º	*T* = 150 K
β = 89.834 (4)º	Needle, colorless
γ = 64.306 (4)º	0.10 × 0.10 × 0.05 mm
*V* = 1968.9 (3) Å^3^	

### Data collection

**Table:**

Bruker Microstar diffractometer	6210 independent reflections
Radiation source: Rotating anode	5656 reflections with *I* > 2σ(*I*)
Monochromator: Helios optics	*R*_int_ = 0.054
Detector resolution: 8.3 pixels mm^-1^	θ_max_ = 68.4º
*T* = 150 K	θ_min_ = 3.4º
ω scans	*h* = −14→14
Absorption correction: multi-scan(SADABS; Sheldrick, 2007)	*k* = −16→16
*T*_min_ = 0.840, *T*_max_ = 0.975	*l* = −16→16
30971 measured reflections	

### Refinement

**Table:**

Refinement on *F*^2^	Secondary atom site location: difference Fourier map
Least-squares matrix: full	Hydrogen site location: inferred from neighbouring sites
*R*[*F*^2^ > 2σ(*F*^2^)] = 0.039	H-atom parameters constrained
*wR*(*F*^2^) = 0.104	*w* = 1/[σ^2^(*F*_o_^2^) + (0.0516*P*)^2^ + 0.5043*P*] where *P* = (*F*_o_^2^ + 2*F*_c_^2^)/3
*S* = 1.05	(Δ/σ)_max_ = 0.001
6210 reflections	Δρ_max_ = 0.24 e Å^−^^3^
476 parameters	Δρ_min_ = −0.18 e Å^−^^3^
66 restraints	Extinction correction: none
Primary atom site location: structure-invariant direct methods	

### Special details

**Table:**

Experimental. X-ray crystallographic data for the title compound were collected from a single-crystal sample, which was mounted on a loop fiber. Data were collected using a Bruker Microstar diffractometer equiped with a platinum-135 CCD detector, Helios optics and a Kappa goniometer. The crystal-to-detector distance was 4.0 cm, and the data collection was carried out in 512 *x* 512 pixel mode. The initial unit-cell parameters were determined by a least-squares fit of the angular setting of strong reflections, collected by a 10.0 degree scan in 33 frames over three different parts of the reciprocal space (99 frames total).Due to geometrical constraints of the instrument and the use of copper radiation, we consistently obtain a data completeness lower than 100% depending on the crystal system and the orientation of the mounted crystal, even with appropriate data collection routines. Typical values for data completeness range from 83–92% for triclinic systems, 85–97% for monoclinic systems and 85–98% for all other crystal systems.
Geometry. All e.s.d.'s (except the e.s.d. in the dihedral angle between two l.s. planes) are estimated using the full covariance matrix. The cell e.s.d.'s are taken into account individually in the estimation of e.s.d.'s in distances, angles and torsional angles; correlations between e.s.d.'s in cell parameters are only used when they are defined by crystal symmetry. An approximate (isotropic) treatment of cell e.s.d.'s is used for estimating e.s.d.'s involving l.s. planes.
Refinement. Refinement of *F*^2^ against ALL reflections. The weighted *R*-factor *wR* and goodness-of-fit *S* are based on *F*^2^ and conventional *R*-factors *R* are based on *F*, with *F* set to zero for negative *F*^2^. The threshold expression of *F*^2^ > σ(*F*^2^) is used only for calculating *R*-factors (gt) *etc*. and is not relevant to the choice of reflections for refinement. *R*-factors based on *F*^2^ are statistically about twice as large as those based on *F*, and *R*- factors based on ALL data will be even larger.

### Fractional atomic coordinates and isotropic or equivalent isotropic displacement parameters (Å^2^)

**Table:**

	*x*	*y*	*z*	*U*_iso_*/*U*_eq_	Occ. (<1)
C1	0.56768 (12)	0.24345 (11)	0.09947 (8)	0.0236 (3)	
H1	0.5210	0.3146	0.0396	0.028*	
C2	0.53181 (12)	0.15288 (11)	0.13794 (9)	0.0250 (3)	
C3	0.60214 (12)	0.04909 (11)	0.22662 (9)	0.0257 (3)	
H3	0.5784	−0.0127	0.2533	0.031*	
C4	0.70461 (12)	0.03441 (11)	0.27598 (8)	0.0241 (3)	
H4	0.7510	−0.0362	0.3363	0.029*	
C5	0.93575 (12)	0.05491 (10)	0.35318 (8)	0.0233 (3)	
H5	0.9415	−0.0209	0.3977	0.028*	
C6	1.02058 (12)	0.08874 (11)	0.36965 (8)	0.0240 (3)	
H6	1.0849	0.0352	0.4264	0.029*	
C7	1.01437 (12)	0.19971 (11)	0.30503 (8)	0.0232 (3)	
C8	0.92079 (12)	0.27689 (10)	0.22061 (8)	0.0227 (3)	
H8	0.9161	0.3518	0.1750	0.027*	
C9	0.72596 (11)	0.31471 (10)	0.11887 (8)	0.0215 (3)	
C10	0.67001 (11)	0.22972 (10)	0.14792 (8)	0.0207 (3)	
C11	0.73907 (11)	0.12504 (10)	0.23588 (8)	0.0207 (3)	
C12	0.83515 (11)	0.24465 (10)	0.20331 (8)	0.0203 (3)	
C13	0.84225 (12)	0.13397 (10)	0.27035 (8)	0.0205 (3)	
C14	0.64303 (12)	0.43903 (10)	0.11943 (8)	0.0239 (3)	
H14A	0.6833	0.4906	0.0972	0.029*	
H14B	0.5704	0.4770	0.0670	0.029*	
C15	0.60497 (13)	0.44029 (11)	0.22170 (9)	0.0298 (3)	
H15A	0.5603	0.3931	0.2431	0.036*	
H15B	0.6767	0.4008	0.2756	0.036*	
C16	0.52763 (13)	0.56797 (12)	0.21393 (9)	0.0314 (3)	
H16A	0.4526	0.6040	0.1650	0.038*	
H16B	0.5692	0.6171	0.1848	0.038*	
C17	0.49743 (14)	0.57588 (12)	0.31602 (10)	0.0342 (3)	
H17A	0.5721	0.5316	0.3674	0.041*	
H17B	0.4478	0.5348	0.3412	0.041*	
C18	0.43181 (14)	0.70437 (12)	0.31007 (10)	0.0340 (3)	
H18A	0.3536	0.7466	0.2637	0.041*	
H18B	0.4780	0.7479	0.2792	0.041*	
C19	0.41195 (13)	0.71020 (12)	0.41468 (10)	0.0317 (3)	
H19A	0.3615	0.6711	0.4434	0.038*	
H19B	0.4898	0.6631	0.4623	0.038*	
C20	0.35339 (14)	0.83778 (12)	0.41154 (10)	0.0332 (3)	
H20A	0.2751	0.8850	0.3646	0.040*	
H20B	0.4035	0.8774	0.3829	0.040*	
C21	0.33548 (15)	0.84006 (13)	0.51746 (10)	0.0375 (3)	
H21A	0.2807	0.8070	0.5438	0.056*	
H21B	0.3019	0.9237	0.5120	0.056*	
H21C	0.4123	0.7909	0.5651	0.056*	
C22	0.75963 (12)	0.33564 (11)	0.01037 (8)	0.0235 (3)	
H22A	0.6853	0.3884	−0.0418	0.028*	
H22B	0.8066	0.3805	0.0002	0.028*	
C23	0.82984 (12)	0.22350 (11)	−0.01143 (9)	0.0265 (3)	
H23A	0.7803	0.1827	−0.0099	0.032*	
H23B	0.9013	0.1665	0.0434	0.032*	
C24	0.86801 (13)	0.25496 (11)	−0.11621 (9)	0.0286 (3)	
H24A	0.7957	0.3114	−0.1703	0.034*	
H24B	0.9151	0.2983	−0.1176	0.034*	
C25	0.94119 (14)	0.14749 (12)	−0.14342 (10)	0.0321 (3)	
H25A	0.8905	0.1109	−0.1521	0.038*	
H25B	1.0078	0.0857	−0.0851	0.038*	
C26	0.99199 (13)	0.18016 (13)	−0.24185 (10)	0.0327 (3)	
H26A	1.0332	0.2269	−0.2368	0.039*	
H26B	1.0524	0.1043	−0.2457	0.039*	
C27	0.90085 (13)	0.25322 (13)	−0.34141 (10)	0.0316 (3)	
H27A	0.8338	0.3226	−0.3337	0.038*	
H27B	0.8689	0.2017	−0.3526	0.038*	
C28	0.95019 (14)	0.30120 (13)	−0.43650 (10)	0.0356 (3)	
H28A	0.9824	0.3525	−0.4255	0.043*	
H28B	1.0168	0.2320	−0.4447	0.043*	
C29	0.85806 (17)	0.37435 (17)	−0.53490 (11)	0.0515 (4)	
H29A	0.8287	0.3229	−0.5483	0.077*	
H29B	0.8942	0.4046	−0.5929	0.077*	
H29C	0.7916	0.4429	−0.5273	0.077*	
B1	1.10946 (14)	0.23555 (12)	0.32833 (10)	0.0240 (3)	
O1	1.18801 (8)	0.17591 (8)	0.41961 (6)	0.0272 (2)	
O2	1.12260 (8)	0.33030 (8)	0.25940 (6)	0.0298 (2)	
C30	1.27671 (12)	0.21973 (11)	0.40392 (9)	0.0276 (3)	
C31	1.20716 (13)	0.34677 (11)	0.31416 (9)	0.0285 (3)	
C32	1.37874 (14)	0.13171 (13)	0.37341 (11)	0.0365 (3)	
H32A	1.4093	0.0500	0.4277	0.055*	
H32B	1.4424	0.1555	0.3645	0.055*	
H32C	1.3508	0.1325	0.3082	0.055*	
C33	1.31708 (15)	0.22104 (14)	0.50430 (10)	0.0392 (4)	
H33A	1.2473	0.2646	0.5304	0.059*	
H33B	1.3673	0.2617	0.4921	0.059*	
H33C	1.3628	0.1376	0.5554	0.059*	
C34	1.13448 (15)	0.44551 (13)	0.35113 (12)	0.0420 (4)	
H34A	1.0804	0.5190	0.2918	0.063*	
H34B	1.1880	0.4629	0.3851	0.063*	
H34C	1.0879	0.4185	0.4004	0.063*	
C35	1.28192 (15)	0.38512 (14)	0.23982 (11)	0.0406 (4)	
H35A	1.3204	0.3251	0.2099	0.061*	
H35B	1.3429	0.3909	0.2770	0.061*	
H35C	1.2309	0.4642	0.1844	0.061*	
B2	0.41847 (15)	0.16668 (14)	0.08183 (11)	0.0293 (3)	
O3	0.34863 (9)	0.26417 (9)	−0.00506 (7)	0.0386 (3)	
O4	0.38022 (11)	0.08195 (11)	0.11362 (8)	0.0570 (4)	
C40	0.27486 (13)	0.12168 (13)	0.04290 (10)	0.0342 (3)	
C41	0.2640 (4)	0.2354 (5)	−0.0494 (4)	0.0418 (13)	0.50
C42	0.1873 (4)	0.1423 (5)	0.1132 (4)	0.0574 (13)	0.50
H42A	0.2108	0.0668	0.1741	0.086*	0.50
H42B	0.1081	0.1689	0.0767	0.086*	0.50
H42C	0.1854	0.2049	0.1351	0.086*	0.50
C43	0.2789 (4)	0.0205 (4)	0.0104 (3)	0.0477 (10)	0.50
H43A	0.3469	−0.0050	−0.0244	0.072*	0.50
H43B	0.2050	0.0517	−0.0370	0.072*	0.50
H43C	0.2874	−0.0490	0.0720	0.072*	0.50
C44	0.3127 (4)	0.2093 (4)	−0.1412 (2)	0.0567 (10)	0.50
H44A	0.2627	0.1858	−0.1721	0.085*	0.50
H44B	0.3944	0.1432	−0.1180	0.085*	0.50
H44C	0.3123	0.2822	−0.1927	0.085*	0.50
C45	0.1452 (3)	0.3420 (3)	−0.0827 (2)	0.0726 (13)	0.50
H45A	0.1174	0.3591	−0.0226	0.109*	0.50
H45B	0.0892	0.3255	−0.1145	0.109*	0.50
H45C	0.1502	0.4123	−0.1332	0.109*	0.50
C51	0.2427 (3)	0.2582 (3)	−0.0269 (3)	0.0366 (14)	0.50
C52	0.3241 (3)	0.0379 (3)	−0.0098 (3)	0.0570 (11)	0.50
H52A	0.3883	0.0493	−0.0433	0.085*	0.50
H52B	0.2611	0.0547	−0.0621	0.085*	0.50
H52C	0.3557	−0.0458	0.0410	0.085*	0.50
C53	0.1733 (4)	0.1101 (4)	0.0946 (3)	0.0471 (10)	0.50
H53A	0.2000	0.0254	0.1416	0.071*	0.50
H53B	0.1063	0.1359	0.0418	0.071*	0.50
H53C	0.1481	0.1613	0.1341	0.071*	0.50
C54	0.2145 (4)	0.2935 (4)	−0.1432 (2)	0.0562 (10)	0.50
H54A	0.1456	0.2829	−0.1588	0.084*	0.50
H54B	0.2834	0.2419	−0.1649	0.084*	0.50
H54C	0.1963	0.3785	−0.1804	0.084*	0.50
C55	0.1435 (3)	0.3527 (3)	0.0019 (3)	0.0493 (8)	0.50
H55A	0.1373	0.4328	−0.0379	0.074*	0.50
H55B	0.1616	0.3326	0.0760	0.074*	0.50
H55C	0.0678	0.3535	−0.0139	0.074*	0.50

### Atomic displacement parameters (Å^2^)

**Table:**

	*U*^11^	*U*^22^	*U*^33^	*U*^12^	*U*^13^	*U*^23^
C1	0.0205 (9)	0.0247 (6)	0.0235 (5)	−0.0087 (6)	0.0025 (5)	−0.0095 (5)
C2	0.0217 (9)	0.0297 (6)	0.0270 (6)	−0.0125 (6)	0.0066 (5)	−0.0141 (5)
C3	0.0251 (9)	0.0273 (6)	0.0287 (6)	−0.0149 (6)	0.0074 (5)	−0.0119 (5)
C4	0.0233 (9)	0.0231 (6)	0.0234 (5)	−0.0101 (6)	0.0043 (5)	−0.0077 (4)
C5	0.0232 (9)	0.0217 (6)	0.0216 (5)	−0.0088 (6)	0.0031 (5)	−0.0073 (4)
C6	0.0185 (8)	0.0260 (6)	0.0222 (5)	−0.0063 (6)	0.0000 (5)	−0.0088 (5)
C7	0.0196 (9)	0.0268 (6)	0.0245 (5)	−0.0096 (6)	0.0052 (5)	−0.0130 (5)
C8	0.0214 (9)	0.0229 (6)	0.0237 (5)	−0.0102 (6)	0.0044 (5)	−0.0092 (4)
C9	0.0193 (8)	0.0226 (6)	0.0219 (5)	−0.0096 (6)	0.0011 (5)	−0.0082 (4)
C10	0.0188 (8)	0.0222 (6)	0.0216 (5)	−0.0084 (6)	0.0046 (5)	−0.0108 (4)
C11	0.0176 (8)	0.0229 (6)	0.0217 (5)	−0.0076 (6)	0.0053 (5)	−0.0113 (4)
C12	0.0173 (8)	0.0212 (6)	0.0204 (5)	−0.0065 (6)	0.0036 (4)	−0.0092 (4)
C13	0.0185 (8)	0.0223 (6)	0.0212 (5)	−0.0081 (6)	0.0054 (5)	−0.0109 (4)
C14	0.0210 (9)	0.0215 (6)	0.0249 (5)	−0.0078 (6)	−0.0007 (5)	−0.0072 (4)
C15	0.0295 (10)	0.0273 (6)	0.0282 (6)	−0.0092 (7)	0.0042 (5)	−0.0113 (5)
C16	0.0303 (10)	0.0291 (7)	0.0314 (6)	−0.0094 (7)	0.0041 (5)	−0.0137 (5)
C17	0.0331 (10)	0.0311 (7)	0.0328 (7)	−0.0087 (7)	0.0051 (6)	−0.0145 (5)
C18	0.0335 (10)	0.0319 (7)	0.0352 (7)	−0.0118 (7)	0.0078 (6)	−0.0159 (5)
C19	0.0289 (10)	0.0318 (7)	0.0338 (6)	−0.0119 (7)	0.0051 (6)	−0.0152 (5)
C20	0.0315 (10)	0.0324 (7)	0.0365 (7)	−0.0134 (7)	0.0073 (6)	−0.0165 (5)
C21	0.0364 (11)	0.0381 (7)	0.0406 (7)	−0.0149 (8)	0.0090 (6)	−0.0213 (6)
C22	0.0226 (9)	0.0247 (6)	0.0211 (5)	−0.0113 (6)	0.0013 (5)	−0.0064 (4)
C23	0.0267 (9)	0.0276 (6)	0.0264 (6)	−0.0136 (6)	0.0061 (5)	−0.0106 (5)
C24	0.0294 (9)	0.0286 (6)	0.0278 (6)	−0.0133 (7)	0.0069 (5)	−0.0115 (5)
C25	0.0323 (10)	0.0300 (7)	0.0331 (6)	−0.0128 (7)	0.0072 (6)	−0.0138 (5)
C26	0.0263 (10)	0.0363 (7)	0.0386 (7)	−0.0124 (7)	0.0104 (6)	−0.0210 (6)
C27	0.0291 (10)	0.0383 (7)	0.0358 (7)	−0.0178 (7)	0.0118 (6)	−0.0209 (6)
C28	0.0365 (10)	0.0407 (8)	0.0377 (7)	−0.0202 (8)	0.0149 (6)	−0.0213 (6)
C29	0.0543 (13)	0.0691 (11)	0.0356 (8)	−0.0324 (10)	0.0137 (7)	−0.0207 (7)
B1	0.0188 (10)	0.0247 (7)	0.0265 (6)	−0.0073 (7)	0.0037 (5)	−0.0114 (5)
O1	0.0236 (6)	0.0306 (4)	0.0277 (4)	−0.0162 (5)	0.0006 (3)	−0.0074 (3)
O2	0.0279 (6)	0.0305 (5)	0.0287 (4)	−0.0156 (5)	−0.0011 (4)	−0.0066 (3)
C30	0.0235 (9)	0.0320 (7)	0.0304 (6)	−0.0174 (7)	0.0021 (5)	−0.0100 (5)
C31	0.0256 (9)	0.0297 (6)	0.0328 (6)	−0.0157 (7)	0.0030 (5)	−0.0112 (5)
C32	0.0249 (10)	0.0343 (7)	0.0475 (8)	−0.0123 (7)	0.0028 (6)	−0.0149 (6)
C33	0.0393 (11)	0.0523 (9)	0.0341 (7)	−0.0302 (8)	0.0022 (6)	−0.0146 (6)
C34	0.0408 (11)	0.0331 (7)	0.0560 (9)	−0.0175 (8)	0.0090 (7)	−0.0214 (7)
C35	0.0439 (11)	0.0407 (8)	0.0404 (7)	−0.0273 (8)	0.0093 (6)	−0.0099 (6)
B2	0.0267 (11)	0.0344 (8)	0.0309 (7)	−0.0167 (8)	0.0060 (6)	−0.0142 (6)
O3	0.0326 (7)	0.0420 (5)	0.0403 (5)	−0.0225 (5)	−0.0063 (4)	−0.0089 (4)
O4	0.0524 (9)	0.0579 (7)	0.0546 (6)	−0.0432 (7)	−0.0193 (5)	0.0065 (5)
C40	0.0263 (10)	0.0481 (8)	0.0398 (7)	−0.0229 (8)	0.0056 (6)	−0.0228 (6)
C41	0.039 (3)	0.049 (2)	0.042 (3)	−0.027 (2)	−0.007 (2)	−0.014 (2)
C42	0.042 (3)	0.094 (4)	0.066 (2)	−0.041 (3)	0.0252 (19)	−0.052 (2)
C43	0.050 (3)	0.057 (2)	0.056 (2)	−0.034 (2)	0.0127 (18)	−0.0325 (17)
C44	0.072 (3)	0.082 (3)	0.0359 (16)	−0.054 (3)	0.0092 (16)	−0.0217 (17)
C45	0.046 (3)	0.055 (2)	0.102 (3)	−0.020 (2)	−0.024 (3)	−0.020 (2)
C51	0.032 (3)	0.057 (3)	0.027 (2)	−0.029 (2)	−0.0033 (17)	−0.0129 (16)
C52	0.042 (3)	0.074 (3)	0.080 (3)	−0.030 (3)	0.014 (2)	−0.053 (2)
C53	0.038 (3)	0.063 (3)	0.053 (2)	−0.033 (2)	0.0109 (17)	−0.0237 (18)
C54	0.046 (3)	0.090 (3)	0.0381 (18)	−0.037 (3)	0.0048 (16)	−0.0247 (18)
C55	0.034 (2)	0.0430 (17)	0.065 (2)	−0.0124 (17)	0.0016 (16)	−0.0215 (15)

### Geometric parameters (Å, °)

**Table:**

C1—C10	1.3744 (17)	C28—C29	1.512 (2)
C1—C2	1.4020 (16)	C28—H28a	0.99
C1—H1	0.95	C28—H28b	0.99
C2—C3	1.4019 (17)	C29—H29a	0.98
C2—B2	1.547 (2)	C29—H29b	0.98
C3—C4	1.3761 (18)	C29—H29c	0.98
C3—H3	0.95	B1—O1	1.3644 (16)
C4—C11	1.3921 (16)	B1—O2	1.3670 (16)
C4—H4	0.95	O1—C30	1.4591 (14)
C5—C6	1.3821 (17)	O2—C31	1.4564 (14)
C5—C13	1.3845 (17)	C30—C32	1.509 (2)
C5—H5	0.95	C30—C33	1.5132 (17)
C6—C7	1.3996 (16)	C30—C31	1.5555 (17)
C6—H6	0.95	C31—C35	1.5115 (18)
C7—C8	1.3961 (17)	C31—C34	1.5145 (19)
C7—B1	1.5500 (18)	C32—H32a	0.98
C8—C12	1.3796 (16)	C32—H32b	0.98
C8—H8	0.95	C32—H32c	0.98
C9—C12	1.5136 (16)	C33—H33a	0.98
C9—C10	1.5226 (15)	C33—H33b	0.98
C9—C14	1.5344 (17)	C33—H33c	0.98
C9—C22	1.5451 (15)	C34—H34a	0.98
C10—C11	1.3981 (16)	C34—H34b	0.98
C11—C13	1.4625 (17)	C34—H34c	0.98
C12—C13	1.4016 (15)	C35—H35a	0.98
C14—C15	1.5208 (16)	C35—H35b	0.98
C14—H14a	0.99	C35—H35c	0.98
C14—H14b	0.99	B2—O4	1.3505 (17)
C15—C16	1.5148 (18)	B2—O3	1.3554 (18)
C15—H15a	0.99	O3—C51	1.420 (4)
C15—H15b	0.99	O3—C41	1.490 (5)
C16—C17	1.5149 (17)	O4—C40	1.4385 (17)
C16—H16a	0.99	C40—C52	1.476 (3)
C16—H16b	0.99	C40—C42	1.486 (4)
C17—C18	1.5165 (18)	C40—C53	1.515 (4)
C17—H17a	0.99	C40—C41	1.553 (6)
C17—H17b	0.99	C40—C43	1.555 (4)
C18—C19	1.5175 (17)	C40—C51	1.588 (5)
C18—H18a	0.99	C41—C45	1.476 (6)
C18—H18b	0.99	C41—C44	1.518 (6)
C19—C20	1.5173 (18)	C42—H42a	0.98
C19—H19a	0.99	C42—H42b	0.98
C19—H19b	0.99	C42—H42c	0.98
C20—C21	1.5173 (18)	C43—H43a	0.98
C20—H20a	0.99	C43—H43b	0.98
C20—H20b	0.99	C43—H43c	0.98
C21—H21a	0.98	C44—H44a	0.98
C21—H21b	0.98	C44—H44b	0.98
C21—H21c	0.98	C44—H44c	0.98
C22—C23	1.5163 (17)	C45—H45a	0.98
C22—H22a	0.99	C45—H45b	0.98
C22—H22b	0.99	C45—H45c	0.98
C23—C24	1.5213 (16)	C51—C55	1.525 (4)
C23—H23a	0.99	C51—C54	1.527 (4)
C23—H23b	0.99	C52—H52a	0.98
C24—C25	1.5176 (18)	C52—H52b	0.98
C24—H24a	0.99	C52—H52c	0.98
C24—H24b	0.99	C53—H53a	0.98
C25—C26	1.5263 (18)	C53—H53b	0.98
C25—H25a	0.99	C53—H53c	0.98
C25—H25b	0.99	C54—H54a	0.98
C26—C27	1.5167 (19)	C54—H54b	0.98
C26—H26a	0.99	C54—H54c	0.98
C26—H26b	0.99	C55—H55a	0.98
C27—C28	1.5232 (17)	C55—H55b	0.98
C27—H27a	0.99	C55—H55c	0.98
C27—H27b	0.99		
			
C10—C1—C2	120.31 (11)	C26—C27—H27B	108.8
C10—C1—H1	119.8	C28—C27—H27B	108.8
C2—C1—H1	119.8	H27A—C27—H27B	107.7
C3—C2—C1	118.56 (11)	C29—C28—C27	113.15 (13)
C3—C2—B2	121.07 (11)	C29—C28—H28A	108.9
C1—C2—B2	120.36 (11)	C27—C28—H28A	108.9
C4—C3—C2	121.69 (11)	C29—C28—H28B	108.9
C4—C3—H3	119.2	C27—C28—H28B	108.9
C2—C3—H3	119.2	H28A—C28—H28B	107.8
C3—C4—C11	118.76 (11)	C28—C29—H29A	109.5
C3—C4—H4	120.6	C28—C29—H29B	109.5
C11—C4—H4	120.6	H29A—C29—H29B	109.5
C6—C5—C13	118.51 (10)	C28—C29—H29C	109.5
C6—C5—H5	120.7	H29A—C29—H29C	109.5
C13—C5—H5	120.7	H29B—C29—H29C	109.5
C5—C6—C7	121.84 (11)	O1—B1—O2	113.40 (11)
C5—C6—H6	119.1	O1—B1—C7	123.37 (11)
C7—C6—H6	119.1	O2—B1—C7	123.22 (11)
C8—C7—C6	118.70 (11)	B1—O1—C30	106.64 (9)
C8—C7—B1	121.23 (10)	B1—O2—C31	107.00 (9)
C6—C7—B1	120.07 (11)	O1—C30—C32	106.27 (10)
C12—C8—C7	120.16 (10)	O1—C30—C33	108.77 (10)
C12—C8—H8	119.9	C32—C30—C33	110.82 (12)
C7—C8—H8	119.9	O1—C30—C31	102.2 (1)
C12—C9—C10	101.25 (9)	C32—C30—C31	113.08 (11)
C12—C9—C14	112.05 (9)	C33—C30—C31	114.90 (11)
C10—C9—C14	112.33 (10)	O2—C31—C35	108.7 (1)
C12—C9—C22	111.71 (10)	O2—C31—C34	106.72 (11)
C10—C9—C22	111.77 (8)	C35—C31—C34	110.49 (11)
C14—C9—C22	107.75 (9)	O2—C31—C30	102.19 (9)
C1—C10—C11	120.07 (10)	C35—C31—C30	114.80 (12)
C1—C10—C9	129.04 (10)	C34—C31—C30	113.20 (11)
C11—C10—C9	110.88 (10)	C30—C32—H32A	109.5
C4—C11—C10	120.61 (11)	C30—C32—H32B	109.5
C4—C11—C13	130.94 (11)	H32A—C32—H32B	109.5
C10—C11—C13	108.45 (10)	C30—C32—H32C	109.5
C8—C12—C13	119.99 (11)	H32A—C32—H32C	109.5
C8—C12—C9	128.97 (10)	H32B—C32—H32C	109.5
C13—C12—C9	111.03 (10)	C30—C33—H33A	109.5
C5—C13—C12	120.78 (11)	C30—C33—H33B	109.5
C5—C13—C11	130.84 (10)	H33A—C33—H33B	109.5
C12—C13—C11	108.38 (10)	C30—C33—H33C	109.5
C15—C14—C9	116.62 (9)	H33A—C33—H33C	109.5
C15—C14—H14A	108.1	H33B—C33—H33C	109.5
C9—C14—H14A	108.1	C31—C34—H34A	109.5
C15—C14—H14B	108.1	C31—C34—H34B	109.5
C9—C14—H14B	108.1	H34A—C34—H34B	109.5
H14A—C14—H14B	107.3	C31—C34—H34C	109.5
C16—C15—C14	111.84 (10)	H34A—C34—H34C	109.5
C16—C15—H15A	109.2	H34B—C34—H34C	109.5
C14—C15—H15A	109.2	C31—C35—H35A	109.5
C16—C15—H15B	109.2	C31—C35—H35B	109.5
C14—C15—H15B	109.2	H35A—C35—H35B	109.5
H15A—C15—H15B	107.9	C31—C35—H35C	109.5
C15—C16—C17	114.23 (10)	H35A—C35—H35C	109.5
C15—C16—H16A	108.7	H35B—C35—H35C	109.5
C17—C16—H16A	108.7	O4—B2—O3	113.26 (12)
C15—C16—H16B	108.7	O4—B2—C2	123.36 (12)
C17—C16—H16B	108.7	O3—B2—C2	123.36 (11)
H16A—C16—H16B	107.6	B2—O3—C51	109.1 (2)
C16—C17—C18	114.02 (11)	B2—O3—C41	108.4 (2)
C16—C17—H17A	108.7	B2—O4—C40	109.04 (11)
C18—C17—H17A	108.7	O4—C40—C52	99.86 (16)
C16—C17—H17B	108.7	O4—C40—C42	98.5 (2)
C18—C17—H17B	108.7	C52—C40—C42	136.4 (2)
H17A—C17—H17B	107.6	O4—C40—C53	114.34 (19)
C17—C18—C19	113.18 (11)	C52—C40—C53	112.0 (2)
C17—C18—H18A	108.9	O4—C40—C41	105.2 (2)
C19—C18—H18A	108.9	C52—C40—C41	95.6 (3)
C17—C18—H18B	108.9	C42—C40—C41	117.0 (3)
C19—C18—H18B	108.9	C53—C40—C41	125.6 (3)
H18A—C18—H18B	107.8	O4—C40—C43	112.7 (2)
C20—C19—C18	114.10 (11)	C42—C40—C43	108.9 (2)
C20—C19—H19A	108.7	C53—C40—C43	84.3 (2)
C18—C19—H19A	108.7	C41—C40—C43	113.6 (3)
C20—C19—H19B	108.7	O4—C40—C51	102.82 (14)
C18—C19—H19B	108.7	C52—C40—C51	114.2 (2)
H19A—C19—H19B	107.6	C42—C40—C51	99.5 (3)
C19—C20—C21	112.63 (11)	C53—C40—C51	112.7 (3)
C19—C20—H20A	109.1	C43—C40—C51	129.56 (19)
C21—C20—H20A	109.1	C45—C41—O3	108.5 (4)
C19—C20—H20B	109.1	C45—C41—C44	111.1 (4)
C21—C20—H20B	109.1	O3—C41—C44	105.3 (4)
H20A—C20—H20B	107.8	C45—C41—C40	115.5 (4)
C20—C21—H21A	109.5	O3—C41—C40	101.6 (3)
C20—C21—H21B	109.5	C44—C41—C40	113.8 (4)
H21A—C21—H21B	109.5	C40—C42—H42A	109.5
C20—C21—H21C	109.5	C40—C42—H42B	109.5
H21A—C21—H21C	109.5	C40—C42—H42C	109.5
H21B—C21—H21C	109.5	C40—C43—H43A	109.5
C23—C22—C9	116.57 (9)	C40—C43—H43B	109.5
C23—C22—H22A	108.2	C40—C43—H43C	109.5
C9—C22—H22A	108.2	O3—C51—C55	106.6 (2)
C23—C22—H22B	108.2	O3—C51—C54	108.4 (4)
C9—C22—H22B	108.2	C55—C51—C54	108.5 (3)
H22A—C22—H22B	107.3	O3—C51—C40	103.10 (18)
C22—C23—C24	111.51 (9)	C55—C51—C40	114.9 (4)
C22—C23—H23A	109.3	C54—C51—C40	114.7 (2)
C24—C23—H23A	109.3	C40—C52—H52A	109.5
C22—C23—H23B	109.3	C40—C52—H52B	109.5
C24—C23—H23B	109.3	H52A—C52—H52B	109.5
H23A—C23—H23B	108	C40—C52—H52C	109.5
C25—C24—C23	115.06 (10)	H52A—C52—H52C	109.5
C25—C24—H24A	108.5	H52B—C52—H52C	109.5
C23—C24—H24A	108.5	C40—C53—H53A	109.5
C25—C24—H24B	108.5	C40—C53—H53B	109.5
C23—C24—H24B	108.5	H53A—C53—H53B	109.5
H24A—C24—H24B	107.5	C40—C53—H53C	109.5
C24—C25—C26	113.59 (10)	H53A—C53—H53C	109.5
C24—C25—H25A	108.8	H53B—C53—H53C	109.5
C26—C25—H25A	108.8	C51—C54—H54A	109.5
C24—C25—H25B	108.8	C51—C54—H54B	109.5
C26—C25—H25B	108.8	H54A—C54—H54B	109.5
H25A—C25—H25B	107.7	C51—C54—H54C	109.5
C27—C26—C25	114.93 (12)	H54A—C54—H54C	109.5
C27—C26—H26A	108.5	H54B—C54—H54C	109.5
C25—C26—H26A	108.5	C51—C55—H55A	109.5
C27—C26—H26B	108.5	C51—C55—H55B	109.5
C25—C26—H26B	108.5	H55A—C55—H55B	109.5
H26A—C26—H26B	107.5	C51—C55—H55C	109.5
C26—C27—C28	113.84 (12)	H55A—C55—H55C	109.5
C26—C27—H27A	108.8	H55B—C55—H55C	109.5
C28—C27—H27A	108.8		
			
C10—C1—C2—C3	0.29 (18)	C8—C7—B1—O2	−13.26 (19)
C10—C1—C2—B2	−178.38 (11)	C6—C7—B1—O2	167.36 (12)
C1—C2—C3—C4	−0.13 (18)	O2—B1—O1—C30	−11.48 (15)
B2—C2—C3—C4	178.53 (12)	C7—B1—O1—C30	168.66 (12)
C2—C3—C4—C11	−0.43 (18)	O1—B1—O2—C31	−8.34 (15)
C13—C5—C6—C7	−0.20 (18)	C7—B1—O2—C31	171.52 (12)
C5—C6—C7—C8	−1.21 (18)	B1—O1—C30—C32	−94.09 (12)
C5—C6—C7—B1	178.19 (11)	B1—O1—C30—C33	146.55 (12)
C6—C7—C8—C12	1.43 (18)	B1—O1—C30—C31	24.66 (12)
B1—C7—C8—C12	−177.97 (11)	B1—O2—C31—C35	144.65 (12)
C2—C1—C10—C11	0.11 (18)	B1—O2—C31—C34	−96.17 (12)
C2—C1—C10—C9	179.01 (11)	B1—O2—C31—C30	22.90 (13)
C12—C9—C10—C1	−179.77 (12)	O1—C30—C31—O2	−28.65 (12)
C14—C9—C10—C1	60.54 (15)	C32—C30—C31—O2	85.16 (12)
C22—C9—C10—C1	−60.72 (16)	C33—C30—C31—O2	−146.25 (11)
C12—C9—C10—C11	−0.78 (12)	O1—C30—C31—C35	−146.12 (10)
C14—C9—C10—C11	−120.48 (11)	C32—C30—C31—C35	−32.31 (14)
C22—C9—C10—C11	118.26 (11)	C33—C30—C31—C35	96.28 (14)
C3—C4—C11—C10	0.83 (17)	O1—C30—C31—C34	85.74 (12)
C3—C4—C11—C13	−179.31 (12)	C32—C30—C31—C34	−160.45 (11)
C1—C10—C11—C4	−0.68 (17)	C33—C30—C31—C34	−31.86 (16)
C9—C10—C11—C4	−179.77 (10)	C3—C2—B2—O4	0.1 (2)
C1—C10—C11—C13	179.43 (10)	C1—C2—B2—O4	178.73 (13)
C9—C10—C11—C13	0.34 (13)	C3—C2—B2—O3	−178.85 (13)
C7—C8—C12—C13	−0.25 (17)	C1—C2—B2—O3	−0.2 (2)
C7—C8—C12—C9	178.66 (11)	O4—B2—O3—C51	10.4 (2)
C10—C9—C12—C8	−178.02 (12)	C2—B2—O3—C51	−170.52 (17)
C14—C9—C12—C8	−58.12 (16)	O4—B2—O3—C41	−10.8 (3)
C22—C9—C12—C8	62.89 (15)	C2—B2—O3—C41	168.2 (2)
C10—C9—C12—C13	0.97 (12)	O3—B2—O4—C40	0.63 (18)
C14—C9—C12—C13	120.87 (11)	C2—B2—O4—C40	−178.41 (12)
C22—C9—C12—C13	−118.12 (10)	B2—O4—C40—C52	107.9 (2)
C6—C5—C13—C12	1.41 (17)	B2—O4—C40—C42	−111.8 (2)
C6—C5—C13—C11	−177.92 (11)	B2—O4—C40—C53	−132.4 (2)
C8—C12—C13—C5	−1.20 (17)	B2—O4—C40—C41	9.2 (3)
C9—C12—C13—C5	179.7 (1)	B2—O4—C40—C43	133.48 (19)
C8—C12—C13—C11	178.26 (10)	B2—O4—C40—C51	−9.9 (2)
C9—C12—C13—C11	−0.83 (13)	B2—O3—C41—C45	137.4 (4)
C4—C11—C13—C5	−0.2 (2)	B2—O3—C41—C44	−103.6 (3)
C10—C11—C13—C5	179.69 (12)	B2—O3—C41—C40	15.2 (3)
C4—C11—C13—C12	−179.57 (12)	O4—C40—C41—C45	−131.7 (3)
C10—C11—C13—C12	0.30 (13)	C42—C40—C41—C45	−23.6 (5)
C12—C9—C14—C15	−52.93 (14)	C43—C40—C41—C45	104.7 (4)
C10—C9—C14—C15	60.26 (13)	O4—C40—C41—O3	−14.5 (3)
C22—C9—C14—C15	−176.21 (10)	C42—C40—C41—O3	93.6 (3)
C9—C14—C15—C16	177.66 (11)	C43—C40—C41—O3	−138.2 (3)
C14—C15—C16—C17	−174.02 (11)	O4—C40—C41—C44	98.2 (3)
C15—C16—C17—C18	173.52 (12)	C42—C40—C41—C44	−153.8 (3)
C16—C17—C18—C19	−174.97 (12)	C43—C40—C41—C44	−25.5 (5)
C17—C18—C19—C20	176.35 (12)	B2—O3—C51—C55	105.8 (3)
C18—C19—C20—C21	−179.74 (12)	B2—O3—C51—C54	−137.6 (3)
C12—C9—C22—C23	63.47 (13)	B2—O3—C51—C40	−15.6 (2)
C10—C9—C22—C23	−49.18 (15)	O4—C40—C51—O3	15.3 (2)
C14—C9—C22—C23	−173.05 (10)	C52—C40—C51—O3	−91.9 (2)
C9—C22—C23—C24	−174.33 (10)	C53—C40—C51—O3	138.9 (2)
C22—C23—C24—C25	178.76 (11)	O4—C40—C51—C55	−100.3 (2)
C23—C24—C25—C26	−172.19 (11)	C52—C40—C51—C55	152.57 (18)
C24—C25—C26—C27	−70.95 (15)	C53—C40—C51—C55	23.3 (3)
C25—C26—C27—C28	171.05 (11)	O4—C40—C51—C54	132.9 (3)
C26—C27—C28—C29	−179.67 (12)	C52—C40—C51—C54	25.8 (5)
C8—C7—B1—O1	166.59 (12)	C53—C40—C51—C54	−103.5 (4)
C6—C7—B1—O1	−12.80 (19)		
